# Prediction of in-hospital mortality risk in cardiac arrest patients using machine learning models: a study based on the MIMIC-IV database with external validation from Yunnan University Affiliated Hospital

**DOI:** 10.1186/s12911-026-03465-6

**Published:** 2026-03-28

**Authors:** Ji Jia, Wei Zhang, Hua-lei Dai, Ying-xia Guan, Zhi-gang Yang, Wei Wei, Xin-jin Zhang, Si-ming Tao

**Affiliations:** 1 Department of Cardiology, Affiliated Hospital of Yunnan University, Kunming, 650021 China; 2 Department of Emergency Medicine, Affiliated Hospital of Yunnan University, Kunming, 650021 China

**Keywords:** Machine learning, Mortality, Cardiac arrest, MIMIC database, XGBoost

## Abstract

**Background:**

Cardiac arrest remains a significant cause of mortality worldwide. Identifying factors associated with in-hospital mortality can improve patient care and outcomes.

**Methods:**

A retrospective analysis was performed, including 1845 patients diagnosed with cardiac arrest from the Medical Information Mart for Intensive Care IV (MIMIC-IV) database and 52 patients from the Affiliated Hospital of Yunnan University (YNU). The study evaluated variables such as demographic characteristics, laboratory test results, and comorbidities. Prediction models for in-hospital mortality following cardiac arrest were constructed using five machine learning algorithms: logistic regression (LR), random forest (RF), extreme gradient boosting (XGBoost), support vector machine (SVM), and light gradient boosting machine (LightGBM). The predictive performance of these models was evaluated using the area under the receiver operating characteristic curve (AUC), as well as metrics including accuracy, precision, recall, and the F1 score.

**Results:**

Among the five machine learning algorithms evaluated, the XGBoost model demonstrated the highest performance. The XGBoost model achieved an AUC of 0.828, with an accuracy of 0.737, a precision of 0.709, an F1 score of 0.713, a recall of 0.717, a positive predictive value (PPV) of 0.709, and a negative predictive value (NPV) of 0.767. External validation of the XGBoost model using data from the Affiliated Hospital of Yunnan University yielded an AUC of 0.845, further supporting its predictive reliability. SHAP (SHapley Additive exPlanations) analysis identified the five variables with the greatest impact on in-hospital mortality in patients with cardiac arrest as mechanical ventilation, age, minimum lactate value, maximum arterial blood gas oxygen partial pressure, and urine output.

**Conclusions:**

The XGBoost model enabled good prediction of in-hospital mortality in ICU admission cardiac arrest patients, which may be widely used in clinical decision-making.

**Clinical trial number:**

Not applicable.

**Supplementary Information:**

The online version contains supplementary material available at 10.1186/s12911-026-03465-6.

## Introduction

Cardiac arrest remains one of the leading causes of death worldwide, with survival rates remaining consistently low despite advances in medical care [1, 2]. Immediate interventions, such as cardiopulmonary resuscitation (CPR) [3–6]and advanced cardiac life support (ACLS) [7–9], are vital to patient survival; however, even with prompt treatment, the prognosis for cardiac arrest remains uncertain. Predicting outcomes for patients who have experienced cardiac arrest is crucial, as it enables medical professionals to make informed decisions about post-resuscitation care [10–12], resource allocation [13, 14], and family counseling [15–17].

Given the complexity of factors influencing cardiac arrest outcomes, machine learning (ML) has emerged as a promising approach to analyze complex, high-dimensional datasets in healthcare, providing opportunities to enhance predictive accuracy and uncover new insights into disease mechanisms [18–20]. By leveraging large-scale, high-quality medical databases, researchers can apply machine learning models to assess factors that may contribute to adverse outcomes following cardiac arrest. Specifically, the Medical Information Mart for Intensive Care (MIMIC) database offers comprehensive, anonymized health data from critically ill patients, making it a valuable resource for investigating patient outcomes [21].

While previous studies have applied machine learning to predict outcomes in cardiac arrest patients, this investigation offers several novel contributions. First, unlike prior works that primarily focused on pre-hospital factors or immediate post-resuscitation variables, our study comprehensively integrates demographic, laboratory, physiological, and intervention data throughout the ICU stay. Second, we utilize external validation from a geographically and demographically distinct population, addressing generalizability limitations common in previous models. Third, our application of SHAP analysis moves beyond mere prediction to provide interpretable insights into the relative importance and interactions of clinical variables, bridging the gap between algorithmic performance and clinical utility. By addressing these key limitations of previous research, this study represents an advancement in both the methodological approach and practical applicability of machine learning for cardiac arrest outcome prediction.

## Materials and methods

### Source of data

The primary data sources for this project included the MIMIC-Ⅳ database and patient admission records from the Department of Cardiology at Yunnan University Affiliated Hospital. The MIMIC-IV is a publicly accessible critical care database developed by the Massachusetts Institute of Technology (MIT), in collaboration with Beth Israel Deaconess Medical Center (BIDMC). It encompasses comprehensive, de-identified clinical data from over 190,000 patients admitted to BIDMC between 2008 and 2019, including more than 45,000 intensive care unit (ICU) admissions. The dataset includes patient demographics, vital signs, laboratory test results, medications, and clinical notes, facilitating diverse research endeavors in critical care medicine. This project received approval from the Institutional Review Boards of MIT and BIDMC, with a waiver of informed consent (Certification No. 43805715, obtained by author Ji Jia). The research data from the Affiliated Hospital of Yunnan University were retrieved and collected by the investigators from the electronic medical record system. This study was approved by the Ethics Committee of the Affiliated Hospital of Yunnan University.

### Study patients

**Fig. 1:**
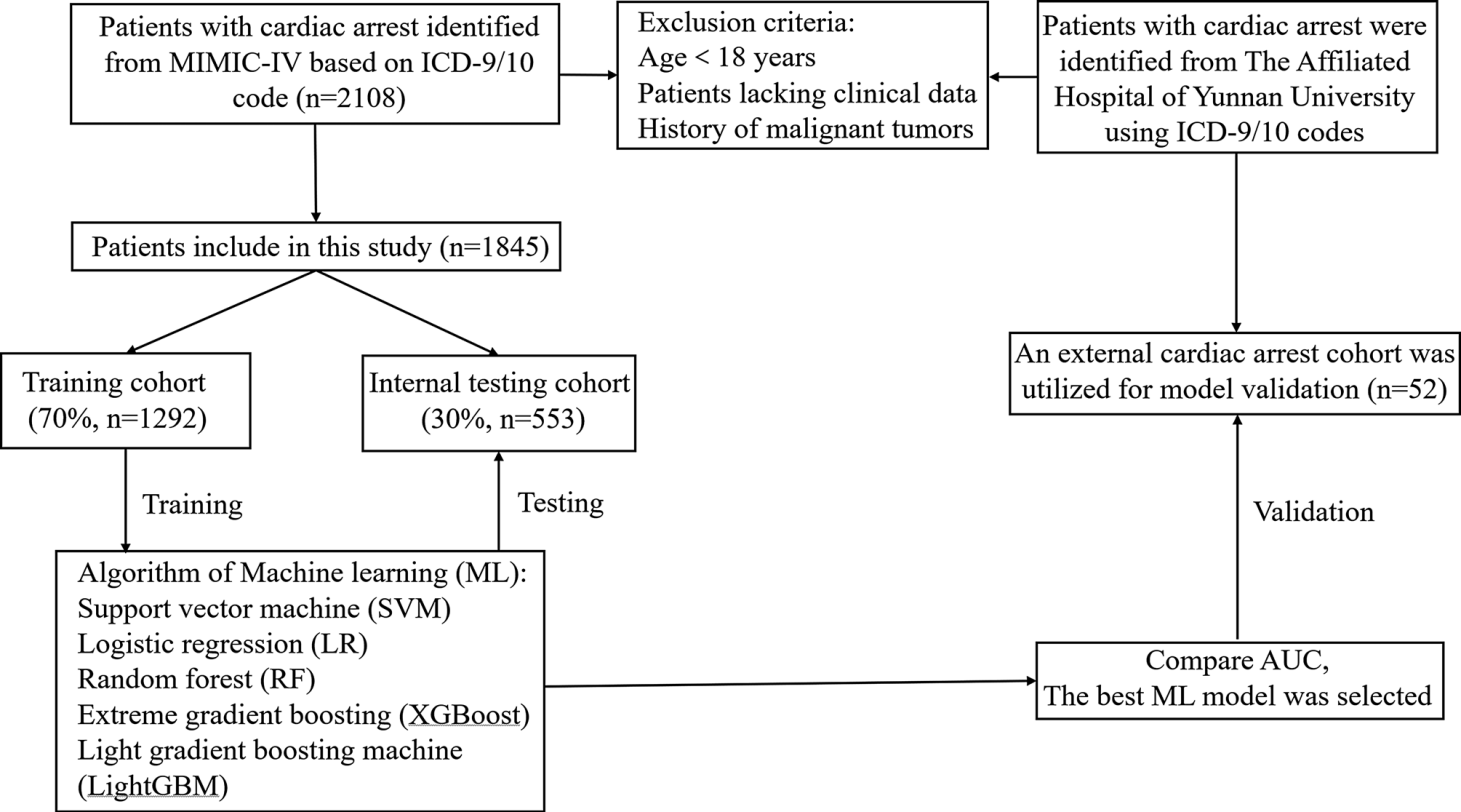
Flowchart of patient selection. ICD-9/10, 9/10th revision of the International Classification of Diseases. MIMIC-IV, Medical Information Mart for Intensive Care IV

All patients from the MIMIC-IV database meeting the inclusion criteria were included in this study. For data from the Affiliated Hospital of Yunnan University, hospitalized patients admitted to the coronary care unit (CCU) of the Department of Cardiology between 2020 and 2024, and meeting the inclusion criteria, were selected. The patient inclusion criteria were as follows: (1) ICD-9/10 diagnosis of cardiac arrest; (2) age greater than 18 years; (3) missing data < 20%; (4) absence of malignant tumors. Additionally, only data from each patient’s first ICU admission was considered for this study. The first ICU admission was considered when a subject had multiple admissions to the ICU. This was a retrospective study utilizing an existing database (MIMIC-IV). All available patients meeting the inclusion criteria were included in the analysis, resulting in 1,845 patients from MIMIC-IV and 52 patients from Yunnan University Affiliated Hospital. No formal sample size calculation was performed as we utilized the maximum available data meeting our inclusion criteria. The screening process is shown in Fig. 1.

### Data extraction

Baseline characteristics, diagnoses, vital signs, and laboratory findings of patients meeting the inclusion criteria were extracted from the MIMIC-IV database using structured query language software PostgreSQL (version 14.5) and Navicat (version 16.0). These data were derived from the following tables: ADMISSIONS, PATIENTS, ICUSTAYS, D_ICD_DIAGNOSES, DIAGNOSES_ICD, LABEVENTS, D_LABITEMS, CHARTEVENTS, D_ITEMS, NOTEEVENTS, and OUTPUTEVENTS. Additionally, data were obtained from the following materialized views: lods, sofa, charlson, ventilation, urine_output, first_day_lab, and first_day_vitalsign. Variables were systematically selected based on clinical relevance and data availability: (1) demographic characteristics: age, gender, and body mass index (BMI); (2) clinical parameters: acute myocardial infarction (AMI), chronic obstructive pulmonary disease (COPD), diabetes, congestive heart failure (CHF), hypertension, and cerebral infarction; (3) laboratory values: creatinine, chloride, glucose, lactate, hemoglobin, platelet count, arterial blood gas oxygen partial pressure (po2), prothrombin time (PT), international normalized ratio (INR), sodium, potassium, blood urea nitrogen (BUN), white blood cell count (WBC), red blood cell count (RBC), red cell distribution width (RDW), calcium, potassium, albumin, creatine kinase MB (CKMB), cardiac troponin T (cTnT), and alanine aminotransferase (ALT); (4) vital signs: the mean levels of heart rate, systolic blood pressure (SBP), diastolic blood pressure (DBP), mean arterial pressure (MAP), temperature, respiratory rate, percutaneous arterial oxygen saturation (SpO2), and first-day urine output; (5) medical scores: Sequential Organ Failure Assessment (SOFA) score, Glasgow Coma Scale (GCS) score, and Logistic Organ Dysfunction System (LODS) score; and (6) medical interventions: ventilation and vasopressor use, including noradrenaline, phenylephrine, epinephrine, dopamine, and dobutamine. Comorbidities were assessed based on recorded International Classification of Diseases (ICD)-9 and ICD-10 codes, and the Charlson Comorbidity Index was calculated. The terms “max” and “min” denote the maximum and minimum values of the included indicators, respectively. For patients from the MIMIC-IV database, maximum and minimum values of laboratory parameters, as well as LODS, SOFA, Charlson, and ventilation scores, were obtained from official materialized views. The specific calculation code can be found in the official documentation (URL: https://github.com/MIT-LCP/mimic-code/). For patients from the Affiliated Hospital of Yunnan University, these values were calculated manually using the same methods as those employed in MIMIC-IV.

### Missing data handling

Missing data were frequently observed in both the MIMIC-IV and Affiliated Hospital of Yunnan University patient datasets. Excluding all patients with missing data could introduce significant research bias; therefore, appropriate handling of missing data is a critical step in data cleaning. In both the MIMIC-IV and Affiliated Hospital of Yunnan University patient datasets, the proportion of missing values for all continuous variables was < 25%, and no missing values were observed for binary variables. The proportion of missing values for different variables is detailed in the supplementary appendix. Missing values were imputed using the R package MICE (version 3.16.0). For continuous variables with a normal distribution, missing values were replaced with the mean of the variable; for skewed data, the median of the variable was used.

### Statistical analysis

Finally, 1845 patients from MIMIC-Ⅳ and 52 patients from Yunnan University Affiliated Hospital were included in the study. In-hospital mortality served as the primary outcome measure, defined as death occurring during the index hospitalization after cardiac arrest. Patients were divided into two groups based on whether they died or survived in hospital, and variables were displayed and compared between groups. Differences in normally distributed data are described as mean ± SD (standard deviation) and were compared by the t-test. In contrast, differences in non-normal data are expressed as IQR (interquartile range) and were compared by a Wilcoxon rank-sum test or chi-squared test. The Shapiro-Wilk test was used to evaluate the normality of continuous variables. Extreme values and outliers were identified using the interquartile range (IQR) method. Values exceeding 1.5 × IQR below the first quartile or above the third quartile were flagged for review. After clinical review, all outliers were retained in the analysis to preserve the natural distribution of clinical data, as extreme values can be clinically meaningful in critical care settings. A two-tailed P value < 0.05 was considered statistically significant. All statistical analyses were performed using R version 4.1.1 (https://www.r-project.org).

This study aimed to develop a machine learning model to predict in-hospital mortality risk in patients with cardiac arrest. Key features were selected using the Boruta algorithm implemented in the R package ‘Boruta’ (version 8.0.0). In the feature selection phase, we employed the Boruta algorithm rather than LASSO regression. The Boruta algorithm identifies all relevant features, rather than producing a minimal optimal subset, which is crucial for clinical prediction models where interpretability is as important as predictive performance. While LASSO regression, through L1 regularization, generates a more concise feature set, it may overlook clinically significant features. Compared to LASSO regression, the feature set generated by the Boruta algorithm offers greater clinical interpretability, facilitating a deeper understanding of the factors influencing patient prognosis and thus, better guiding clinical decision-making. Initially, the Boruta feature selection algorithm was applied to identify features related to the outcome variable. Variables were assigned Z-scores, with those exceeding the shadow features’ Z-score marked as “important,” while variables with significantly lower Z-scores were labeled “unimportant” and subsequently removed from the feature set. This yielded the optimized feature set for model construction. Predictive models were then constructed using five distinct machine learning algorithms—logistic regression (LR), XGBoost, random forest (RF), LightGBM, and support vector machine (SVM)—based on the previously selected feature set. To facilitate model development and internal validation, the MIMIC-IV patient data were randomly divided into training and validation cohorts, with a 70:30 allocation. To prevent overfitting, ten-fold cross-validation and grid search methods were employed for model training. Machine learning analyses used R packages including xgboost (version 1.7.8.1), randomForest (version 4.7–1.1), e1071 (version 1.7–16), caret (version 6.0–94) and lightgbm (version 4.5.0), all under open-source licenses available at their respective repositories. Model performance was assessed by comparing the area under the curve (AUC) of the receiver operating characteristic (ROC) for each model, with the model achieving the highest AUC chosen as the best-performing model for each algorithm. Finally, model validity was evaluated on the test set using AUC, accuracy, precision, recall, F1 score, positive predictive value (PPV) and negative predictive value (NPV). The model with the best predictive performance was selected for external validation using data from patients in the Department of Cardiology at the Affiliated Hospital of Yunnan University.

## Results

### Patients and clinical characteristics

Participants from the MIMIC-VI database were classified as survivors (*n* = 814) or non-survivors (*n* = 1845). The baseline characteristics of these groups are presented in Table 1. Non-survivors were older and demonstrated higher LODS and SOFA scores compared to survivors. Statistically significant differences (*p* < 0.05) were observed between the survivor and non-survivor groups in various parameters, including age, sex, the prevalence of MI, HF, hypertension, and mean values of temperature, heart rate, SBP, DBP, MBP, respiratory rate, and SpO2. Additionally, significant differences were noted in laboratory parameters such as hematocrit_min, hemoglobin_max, hemoglobin_min, wbc_max, wbc_min, platelets_max, platelets_min, glucose_max, glucose_min, bun_max, bun_min, albumin_max, albumin_min, creatinine_max, creatinine_min, sodium_max, potassium_max, chloride_max, calcium_max, calcium_min, inr_max, inr_min, pt_max, pt_min, ph_max, ph_min, lactate_max, lactate_min, alt_max, alt_min, ast_max, ast_min, urine output, as well as levels of cTnT and CK-MB. Non-survivors used vasopressor more often than survivors (35.4% vs. 20.9%, *p* < 0.001). Detailed patient characteristics for the cohort from Yunnan University Affiliated Hospital can be found in Supplementary Table S1.

**Table 1:** Characteristic at baseline between survivors and non-survivors group in MIMIC-IV

Variables	Total (n=1845)	Survivors (n=814)	Non-survivors (n=1031)	P-value
age	67.5 (53.9, 82.3)	66.0 (56.4, 77.4)	71.8 (58.5, 82.3)	＜0.001
male	1099 (59.6)	510 (62.7)	589 (57.1)	0.016
BMI	29.0 ± 8.2	29.4 ± 8.8	28.8 ± 7.7	0.193
**Comorbidities**				
MI	541 (29.3)	274 (33.7)	267 (25.9)	＜0.001
hypertension	1077 (54.6)	479 (58.8)	528 (51.2)	0.001
diabetes	706 (38.3)	316 (38.8)	390 (37.8)	0.663
HF	829 (44.9)	414 (50.9)	415 (40.3)	＜0.001
COPD	141 (7.6)	72 (8.8)	69 (6.7)	0.084
cerebral infarction	340 (18.4)	157 (19.3)	183 (17.7)	0.398
**Vital** **signs**				
temperature_mean	36.7 (36.3, 37.0)	36.8 (36.5, 37.1)	36.6 (36.0, 37.0)	＜0.001
heart_rate_mean	82.2 (71.5, 95.7)	80.1 (69.7, 91.8)	84.9 (73.0, 99.1)	＜0.001
sbp_mean	112.0 (103.2, 122.7)	113.9 (105.5, 125.2)	110.1 (101.1, 121.3)	＜0.001
dbp_mean	61.3 (53.6, 68.7)	62.3 (55.3, 68.7)	60.4 (52.3, 68.8)	＜0.001
mbp_mean	80.6 (73.5, 87.6)	81.1 (73.5, 87.6)	78.3(71.3, 86.2)	＜0.001
resp_rate_mean	20.0 (17.3, 23.4)	19.0 (16.8, 22.1)	21.0 (17.8, 24.5)	＜0.001
spo2_mean	97.4 (95.7, 98.9)	97.6 (96.3, 98.9)	97.2 (94.9, 98.8)	＜0.001
**Laboratory** **results**				
hematocrit_max	35.4 (30.8, 41.2)	35.9 (31.1, 41.5)	35.0 (30.6, 41.0)	0.133
hematocrit_min	30.5 (25.5, 36.2)	31.0 (25.8, 36.7)	30.1 (25.2, 35.8)	0.046
hemoglobin_max	11.4 (9.8, 13.5)	11.8 (10.0, 13.7)	11.3 (9.6, 13.3)	0.001
hemoglobin_min	9.9 (8.1, 12.0)	10.2 (8.4, 12.3)	9.6 (7.9, 11.7)	＜0.001
wbc_max	15.1 (10.6, 20.6)	10.3 (7.1, 14.5)	16.1 (11.1, 21.5)	＜0.001
wbc_min	9.9 (7.1, 13.6)	9.5 (7.1, 12.7)	10.3 (7.1, 14.5)	0.006
platelets_max	217.0 (161.0, 290.0)	218.0 (166.0, 286.0)	217.0 (157.0, 292.5)	0.192
platelets_min	166.0 (119.0, 227.0)	171.5 (128.3, 230.0)	160.0 (108.0, 225.0)	＜0.001
glucose_max	195.0 (142.0, 290.0)	195.0 (142.0, 290.0)	210.0 (152.0, 308.0)	＜0.001
glucose_min	120.0 (97.0, 150.0)	116.0 (96.0, 138.0)	125.0 (98.0, 161.0)	＜0.001
bun_max	29.0 (19.0, 46.0)	25.0 (17.0, 39.0)	32.0 (22.0, 50.0)	＜0.001
bun_min	23.0 (15.0, 39.0)	20.0 (14.0, 32.0)	26.0 (16.0, 42.0)	＜0.001
albumin_max	3.2 (2.7, 3.7)	3.3 (2.9, 3.8)	3.1 (2.6, 3.6)	＜0.001
albumin_min	3.1 (2.6, 3.5)	3.2 (2.7, 3.7)	3.0 (2.4, 3.4)	＜0.001
creatinine_max	1.5 (1.0, 2.5)	1.3 (0.9, 2.0)	1.7 (1.2, 2.7)	＜0.001
creatinine_min	1.2 (0.8, 1.9)	1.0 (0.7, 1.6)	1.3 (0.9, 2.1)	＜0.001
sodium_max	141.0 (138.0, 144.0)	140.0 (138.0, 143.0)	141.0 (137.0, 144.0)	0.001
sodium_min	137.0 (134.0, 140.0)	137.0 (134.0, 140.0)	137.0 (133.0, 140.0)	0.927
potassium_max	4.6 (4.2, 5.4)	4.6 (4.2, 5.2)	4.7 (4.3, 5.5)	＜0.001
potassium_min	3.8 (3.4, 4.2)	3.8 (3.5, 4.2)	3.8 (3.4, 4.3)	0.109
chloride_max	106.0 (102.0, 110.0)	106.0 (102.0, 109.0)	106.0 (101.0, 111.0)	0.014
chloride_min	101.0 (97.0, 105.0)	102.0 (98.0, 105.0)	101.0 (97.0, 106.0)	0.479
calcium_max	8.7 (8.1, 9.2)	8.7 (8.3, 9.2)	8.6 (8.0, 9.2)	＜0.001
calcium_min	8.0 (7.3, 8.6)	8.1 (7.5, 8.6)	7.8 (7.2, 8.5)	＜0.001
inr_max	1.4 (1.2, 2.0)	1.3 (1.1, 1.6)	1.6 (1.3, 2.3)	＜0.001
inr_min	1.2 (1.1, 1.5)	1.2 (1.1, 1.4)	1.3 (1.1, 1.7)	＜0.001
pt_max	15.5 (13.2, 21.9)	14.3 (12.6, 17.9)	17.0 (13.9, 24.7)	＜0.001
pt_min	13.8 (12.2, 16.7)	13.0 (11.8, 15.2)	14.4 (12.6, 18.9)	＜0.001
ph_max	7.4 (7.3, 7.5)	7.4 (7.4, 7.5)	7.4 (7.3, 7.5)	＜0.001
ph_min	7.3 (7.1, 7.4)	7.3 (7.2, 7.4)	7.2 (7.1, 7.3)	＜0.001
lactate_max	4.0 (2.1, 7.5)	3.2 (1.7, 5.5)	4.8 (2.6, 8.8)	＜0.001
lactate_min	1.8 (1.2, 3.4)	1.5 (1.1, 2.2)	2.2 (1.4, 4.4)	＜0.001
po2_max	228.0 (129.5, 342.0)	239.0 (152.0, 368.0)	215.0 (120.0, 330.0)	＜0.001
po2_min	82.0 (65.0, 115.0)	84.0 (68.0, 119.0)	81.0 (63.0, 112.0)	0.01
alt_max	73.0 (32.0, 233.0)	56.0 (27.0, 165.0)	89.0 (36.0, 285.5)	＜0.001
alt_min	52.0 (26.0, 150.0)	45.0 (23.0, 110.0)	63.0 (30.0, 182.5)	＜0.001
ast_max	116.0 (48.0, 359.0)	80.0 (40.0, 230.8)	161.0 (58.0, 483.5)	＜0.001
ast_min	78.0 (38.0, 212.0)	56.5 (33.0, 150.0)	96.0 (45.0, 298.0)	＜0.001
cTnT	0.10 (0.03, 0.42)	0.09 (0.03, 0.31)	0.13 (0.04, 0.51)	＜0.001
ckmb	5.0 (3.0, 15.0)	4.0 (2.0, 10.0)	6.0 (3.0, 19.0)	＜0.001
**Score** **system**				
SOFA	7 (4, 10)	5 (3, 9)	8 (5, 11)	＜0.001
GCS	15 (14, 15)	15 (14, 15)	15 (14, 15)	0.147
LODS	7 (4, 10)	7 (4, 10	8 (5, 10)	＜0.001
Charlson	5 (3, 8)	6 (3, 8)	6 (4, 8)	＜0.001
**Others**				
urineoutput	1075 (413, 1895)	1450 (818, 2260)	810 (242, 1517)	＜0.001
ventilation	3.4 (1.1, 9.1)	5.1 (1.8, 14.8)	2.7 (0.8, 6.4)	＜0.001
vasopressor	535 (29.0)	170 (20.9)	365 (35.4)	＜0.001

Medical conditions were defined based on ICD-9 codes. The terms mean, minimum, or maximum refer to the average, highest, or lowest recorded level of a parameter on the first day of ICU admission. Abbreviations used are as follows: BMI, body mass index; MI, myocardial infarction; HF, heart failure; COPD, chronic obstructive pulmonary disease; WBC: white blood cell count; SpO₂, pulse oxygen saturation; po2, arterial blood gas oxygen partial pressure; INR, international normalized ratio; PT, prothrombin time; BUN, blood urea nitrogen; WBC, white blood cell count; SBP, systolic blood pressure; DBP, diastolic blood pressure; MBP, mean blood pressure; cTnT: cardiac troponin T; CKMB: creatine kinase isoenzyme MB; ALT: alanine aminotransferase; AST: aspartate aminotransferase; SOFA, Sequential Organ Failure Assessment; GCS, Glasgow Coma Scale; LODS, Logistic Organ Dysfunction System. Ventilation: Duration of ventilator use during hospitalization (measured in hours)

### Feature selection

**Fig. 2:**
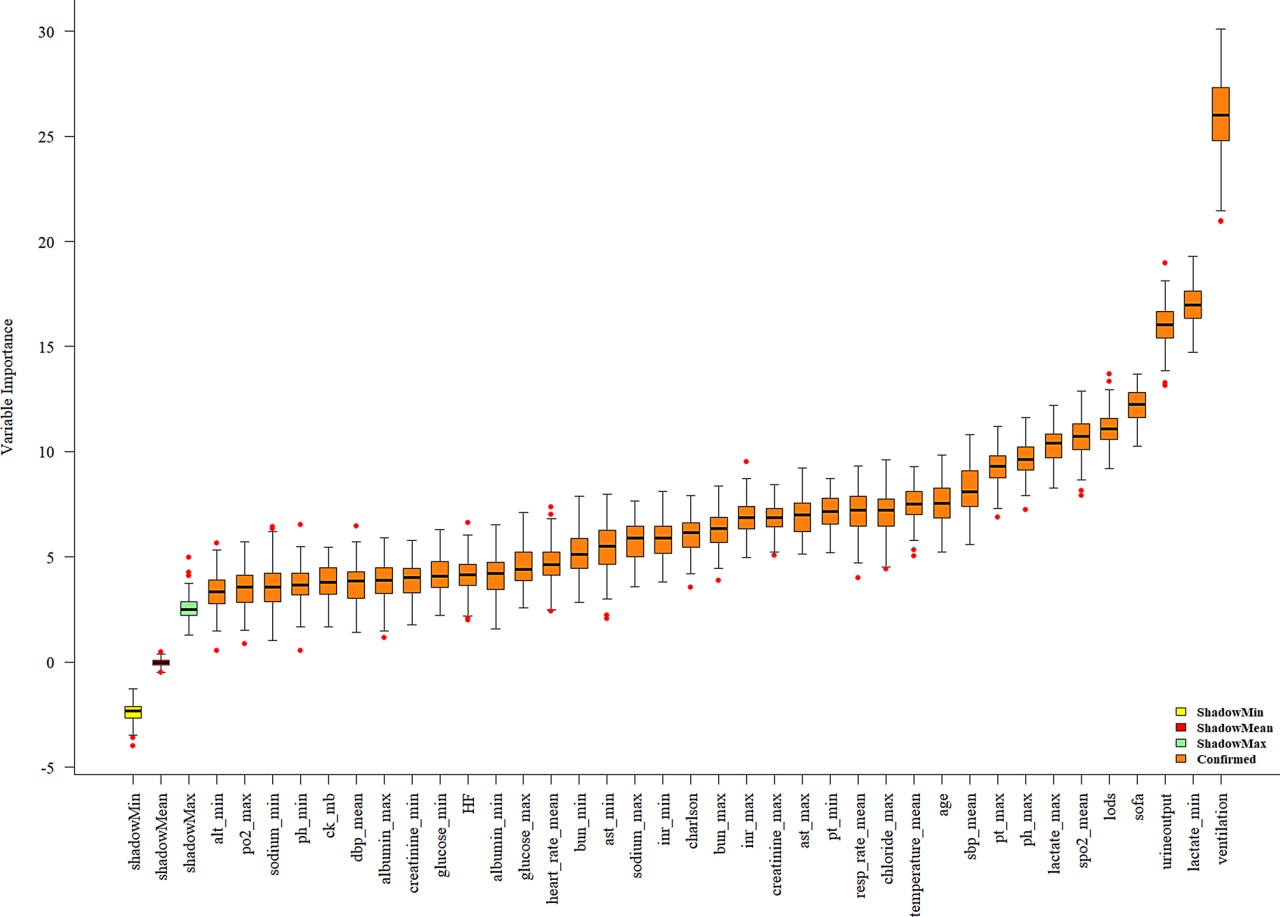
Importance scores for patient feature selection following cardiac arrest. The term “shadow” refers to the shadow feature, while “confirmed” denotes the features identified as significant

Feature selection was performed using the Boruta algorithm. Shadow features were constructed by combining the original true features with randomly permuted versions of these features to create a feature matrix for training. The feature importance scores of the shadow features served as reference benchmarks, allowing the Boruta package to identify a set of features relevant to the dependent variable from the true features. This selection process resulted in a feature set comprising 37 important variables. A total of 37 variables closely associated with in-hospital mortality following cardiac arrest were identified: age, heart_rate_mean, sbp_mean, dbp_mean, temperature_mean, spo2_mean, resp_rate_mean, albumin_min, albumin_max, bun_min, bun_max, chloride_max, creatinine_min, creatinine_max, glucose_min, glucose_max, sodium_min, sodium_max, alt_min, ast_min, ast_max, pt_min, pt_max, inr_min, inr_max, lactate_min, lactate_max, ph_min, ph_max, po2_max, urineoutput, ck_mb, sofa, charlson, lods, ventilation, and HF (Fig. 2).

### Model comparison

**Fig. 3:**
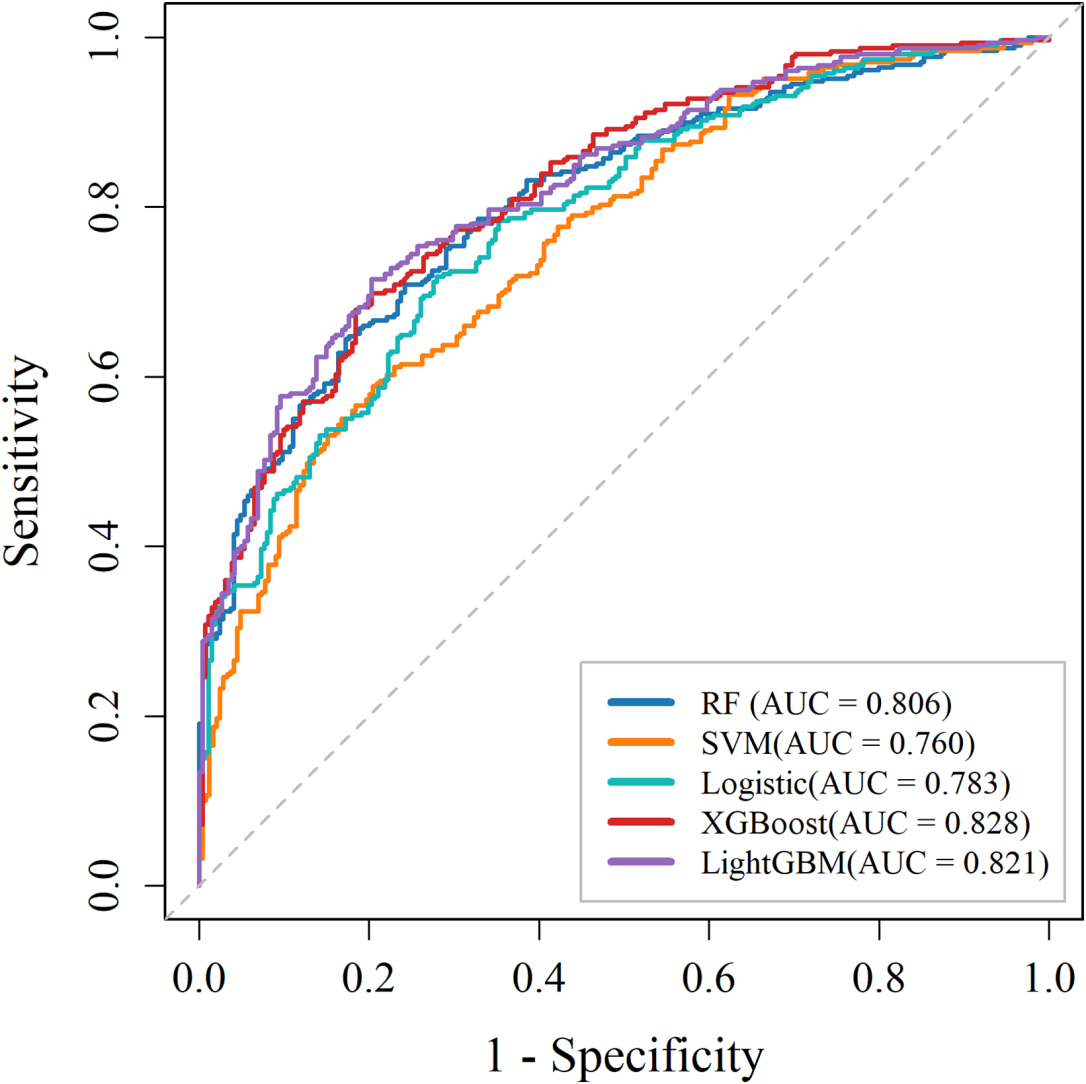
Performance evaluation for five machine learning algorithms with ROC curves

Table 2 presents the performance of five machine learning models for predicting in-hospital mortality among patients with cardiac arrest. The evaluation metrics include the AUC, accuracy, precision, F1 score, recall, PPV and NPV. Among the five machine learning algorithms evaluated, XGBoost demonstrated superior performance across all metrics, achieving the highest AUC (0.828) and accuracy (0.737). LightGBM exhibited comparable results, with an AUC of 0.821 and the highest precision (0.726). The RF model also showed robust performance, with an AUC of 0.806 and a noteworthy NPV of 0.748. Conversely, the SVM model demonstrated relatively lower predictive capability, with an AUC of 0.760 and the lowest recall (0.557). LR achieved intermediate results, with an AUC of 0.783 and accuracy of 0.717. These findings suggest that ensemble learning methods, such as XGBoost and LightGBM, outperform traditional machine learning models in predicting in-hospital mortality risk in this patient population (Fig. 3).

**Table 2:** Performance of the machine learning model in the internal testing cohort

Models	AUC	Accuracy	Precision	F1 score	Recall	PPV	NPV
RF	0.806 (0.771, 0.842)	0.732 (0.694, 0.768)	0.710 (0.649, 0.766)	0.686 (0.636, 0.730)	0.664 (0.598, 0.718)	0.710 (0.649, 0.766)	0.748 (0.649, 0.766)
SVM	0.760 (0.720, 0.799)	0.687 (0.645, 0.722)	0.677 (0.614, 0.737)	0.611 (0.544, 0.659)	0.557 (0.485, 0.610)	0.677 (0.614, 0.737)	0.693 (0.611, 0.743)
LR	0.783 (0.746, 0.820)	0.717 (0.680, 0.754)	0.711 (0.649, 0.767)	0.680 (0.631, 0.726)	0.651 (0.591, 0.711)	0.711 (0.649, 0.767)	0.722 (0.670, 0.768)
XGBoost	0.828 (0.786, 0.854)	0.737 (0.712, 0.784)	0.709 (0.648, 0.767)	0.713 (0.631, 0.754)	0.717 (0.654, 0.764)	0.709 (0.648, 0.767)	0.767 (0.670, 0.783)
LightGBM	0.821 (0.782, 0.850)	0.737 (0.698, 0.773)	0.726 (0.668, 0.782)	0.707 (0.660, 0.749)	0.690 (0.631, 0.743)	0.726 (0.668, 0.782)	0.745 (0.694, 0.792)

The AUC, accuracy, precision, F1 score, recall, PPV, NPV, and 95% CIs were calculated for the RF, SVM, LR, XGBoost and LightGBM models. Abbreviations: RF: random forest; SVM: support vector machine; LR: logistic regression; XGBoost: extreme gradient boosting; LightGBM: light gradient boosting machine; PPV: positive predictive value; NPV: negative predictive value; AUC: area under the curve

**Fig. 4:**
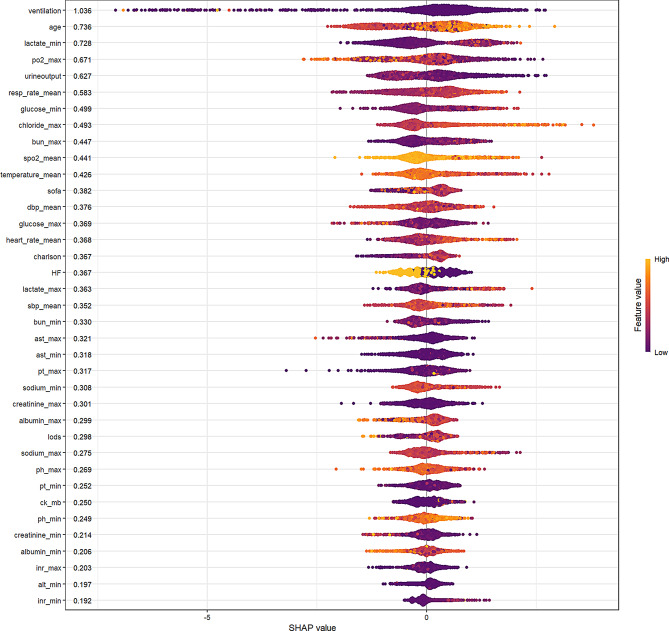
Feature importance was assessed using Shapley Additive Explanations (SHAP) values. Features are ranked based on the sum of SHAP value magnitudes across all samples. The color gradient represents feature values, with yellow indicating higher values and purple indicating lower values. The x-axis quantifies the impact on the model output, with positive contributions on the right and negative contributions on the left. For instance, considering the feature “age,” a greater concentration of yellow points on the right suggests that higher age scores are associated with increased prediction scores

The SHAP (Shapley additive explanations) analysis of the XGBoost model provides a detailed assessment of feature importance in predicting patient outcomes. Ventilation was identified as the most influential predictor, with the highest absolute SHAP value (1.036), followed by age (0.736) and minimum lactate levels (0.728) as the second and third most significant features, respectively. Other notable physiological parameters include maximum po2 (0.671), mean respiratory rate (0.583), mean SpO₂ (0.441), and mean temperature (0.426). Significant laboratory variables include minimum glucose (0.499), maximum chloride levels (0.493), and maximum BUN (0.447). Additionally, hemodynamic parameters such as mean heart rate (0.368) and various blood pressure measurements, along with the SOFA score (0.382), were observed to have a moderate impact on the predictions. The color gradient in the visualization, ranging from purple (indicating low values) to yellow (indicating high values), highlights the distribution of SHAP values for each feature, offering insights into their directional influence on the model’s outcomes. This analysis provides a quantitative framework for understanding the relative importance of clinical parameters in the XGBoost model, with ventilation, age, and lactate levels forming the top tier of predictors in the hierarchy of feature importance. (Fig. 4)

**Fig. 5:**
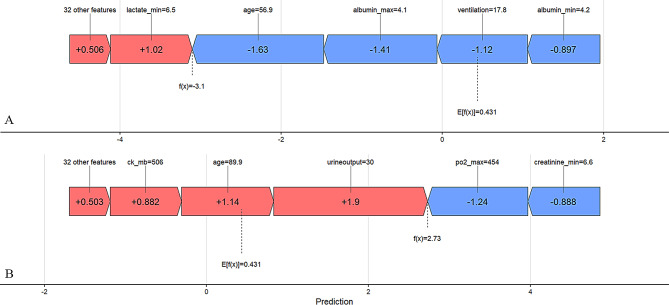
The predicted outcomes for two specific instances are presented. Red bars denote risk factors, while blue bars represent protective factors. The length of the bars indicates the relative importance of the corresponding feature values, with longer bars reflecting greater significance

Figure 5 shows the SHAP feature contribution plots for patients predicted to survive and those predicted to die. In panel A, the prediction of survival can be explained by key factors such as relatively younger age, shorter duration of ventilator use, and normal albumin levels. In panel B, the prediction of death can be attributed to key factors such as lower urine output and older age.

### External validation of the model

**Fig. 6:**
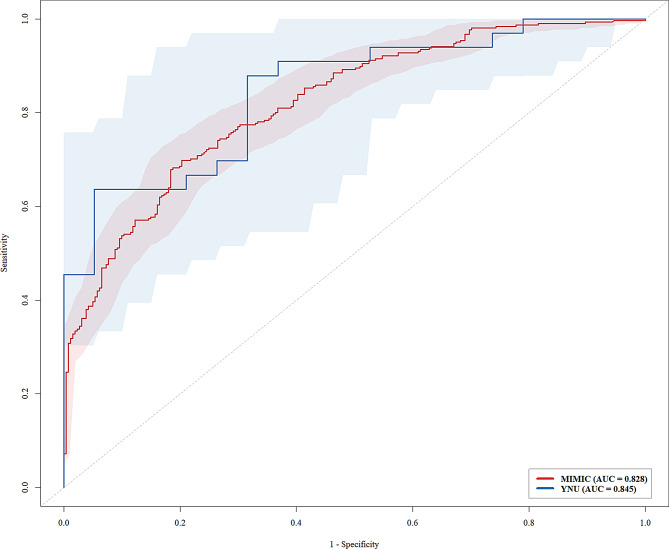
ROC curve of the XGBoost model for predicting in-hospital mortality among cardiac arrest patients using data from the MIMIC-IV database and the Affiliated Hospital of Yunnan University

As discussed earlier, among the five machine learning methods evaluated, XGBoost demonstrated the best overall predictive performance. To validate the model, we conducted external validation using data from patients diagnosed with cardiac arrest at the Affiliated Hospital of Yunnan University, in addition to internal validation (Fig. 6). The AUC for internal and external validation were 0.828 and 0.845, respectively, with no statistically significant difference observed between them (*p* = 0.6544).

## Discussion

This study demonstrates the successful application of machine learning approaches in predicting in-hospital mortality among cardiac arrest patients, with XGBoost emerging as the most effective predictive model. To elucidate the contribution of individual features, SHAP analysis was employed, revealing that the most important variables influencing model predictions included ventilator duration, lactate levels, age, arterial blood gas oxygen partial pressure, and urine output. Each of these variables provides critical insights into the patient’s physiological state and is closely linked to clinical outcomes [22–25]. Mechanical ventilation is a critical intervention for patients with respiratory failure following cardiac arrest. However, prolonged mechanical ventilation is associated with an increased risk of ventilator-associated pneumonia (VAP), which can lead to sepsis and multiorgan failure, ultimately contributing to higher mortality rates [26, 27]. In our study, mechanical ventilation was identified as the most influential predictor of in-hospital mortality, underscoring the importance of minimizing ventilator dependency and implementing strategies to prevent VAP in this patient population. Lactate levels were another key predictor, serving as a marker of systemic hypoperfusion and metabolic stress. Elevated lactate levels, another critical predictor, reflect significant tissue hypoperfusion and oxygen debt. In cardiac arrest patients, persistent lactate elevation despite resuscitation efforts signals ongoing cellular distress and metabolic derangement, often preceding multi-organ dysfunction [28–30]. The relationship between lactate clearance and survival has been well-documented, with slower clearance patterns associated with poorer outcomes [29, 31, 32]. Age is a well-established predictor of mortality in cardiac arrest patients. Older age is associated with declining physiological reserve, including reduced cardiac output, decreased renal function, and impaired immune response. These age-related changes make older patients more vulnerable to the stressors of cardiac arrest and the subsequent critical care environment, increasing their risk of adverse outcomes [33–35]. Arterial blood gas oxygen partial pressure provides crucial information about oxygenation efficiency and pulmonary function, with lower levels suggesting compromised gas exchange, which is particularly relevant in post-cardiac arrest patients who often experience respiratory dysfunction [36–38]. Lastly, urine output serves as an important indicator of renal function and hemodynamic stability, with oliguria often signaling impaired organ perfusion and impending multiorgan failure [39]. Interestingly, maximum chloride levels emerged as an important predictor. While not traditionally emphasized in cardiac arrest management, chloride disturbances may reflect acid-base imbalances, renal dysfunction, or aggressive fluid resuscitation with chloride-rich solutions [40, 41]. Maximum chloride levels were identified as an important feature in our model, suggesting that higher chloride levels are associated with increased mortality. Hyperchloremia, or high serum chloride concentration, is a common electrolyte abnormality in critically ill patients and has been linked to poor outcomes, including increased mortality. The mechanisms underlying this association are multifactorial and may include the development of metabolic acidosis, fluid overload from chloride-rich intravenous fluids, and acute kidney injury [42, 43]. In our cohort, maximum chloride levels likely reflect the peak chloride concentration during the hospital stay, which could be influenced by fluid resuscitation practices. Therefore, careful management of fluid therapy and monitoring of chloride levels are essential to mitigate the risks associated with hyperchloremia. The identification of key predictive factors through SHAP analysis provides clinicians with actionable insights for risk stratification and patient management.

To ensure the generalizability of the XGBoost model, external validation was conducted using a dataset from Yunnan University Affiliated Hospital. The model performed robustly in this independent cohort, confirming its applicability across diverse patient populations and clinical settings. This consistency across datasets underscores the reliability and clinical relevance of these predictors in mortality risk stratification.

The findings of this study highlight the potential of machine learning models, particularly XGBoost, to enhance decision-making in critical care by enabling early and accurate identification of high-risk cardiac arrest patients [44]. The predictive output of the XGBoost model can be integrated into clinical workflows to refine risk stratification and inform post-resuscitation management of cardiac arrest patients. Clinicians can employ the model’s probability score (range 0 to 1) to categorize patients into distinct risk strata: low-risk (score < 0.3), intermediate-risk (0.3–0.7), and high-risk (score > 0.7). For high-risk patients, priority should be given to aggressive interventions targeting modifiable predictors identified through SHAP analysis, including optimization of mechanical ventilation to minimize duration, meticulous monitoring of lactate levels to mitigate hypoperfusion, and ensuring adequate urine output via fluid management or renal support. Early consultation with multidisciplinary critical care teams may further benefit these patients. Intermediate-risk patients warrant frequent reassessment, encompassing serial lactate measurements and hemodynamic monitoring, to detect potential deterioration. Conversely, low-risk patients may require standard monitoring and could be considered for earlier de-escalation of intensive interventions. To facilitate clinical implementation, the model should be embedded within electronic health records (EHRs) to provide real-time risk scores upon ICU admission, accompanied by context-specific alerts (e.g., “High mortality risk: escalate hemodynamic support”) and actionable recommendations. Notably, model outputs are intended to augment, not supplant, clinical judgment, with decision-making incorporating patient-specific factors such as comorbidities, care goals, and family preferences. Prospective validation of this framework in dynamic clinical settings is crucial to refine its utility and ensure alignment with resource availability and institutional protocols.

This study underscores the potential of machine learning models, notably XGBoost, to enhance risk stratification and clinical decision-making for cardiac arrest patients within critical care environments. By elucidating key mortality predictors and generating actionable risk categories, the model provides clinicians with a data-driven instrument to personalize post-resuscitation interventions. While the proposed framework emphasizes pragmatic integration into clinical workflows, its real-world efficacy necessitates validation through prospective studies evaluating its impact on patient outcomes, resource utilization, and clinician adherence. Subsequent investigations should prioritize refining model interpretability, broadening validation across heterogeneous patient populations, and developing dynamic update mechanisms to incorporate real-time physiological data. Furthermore, ethical considerations, including mitigation of algorithmic bias and ensuring equitable access to predictive tools, must be rigorously addressed. Ultimately, the synergistic application of robust predictive models with clinical expertise and patient-centered care represents a promising avenue for advancing resuscitation science and optimizing survival rates in this high-risk patient cohort.

### Limitations of the study

A major strength of this study is its use of a large, well-curated database for model development and the inclusion of an independent dataset for validation, ensuring robust and generalizable findings. The incorporation of diverse clinical variables and rigorous statistical analysis further strengthens the reliability of our results. However, several limitations warrant consideration. First, the retrospective nature of the study may introduce bias, as it relies on the availability and accuracy of recorded data. Second, despite efforts to address missing data through imputation, residual data quality issues may still impact the models’ performance. Finally, the relatively small sample size of the external validation cohort may limit the applicability of the findings to broader populations.

## Conclusion

This study demonstrates that: (1) Machine learning models, particularly XGBoost, can effectively predict in-hospital mortality in cardiac arrest patients. (2) Key clinical predictors identified through SHAP analysis offer valuable insights for risk stratification. (3) External validation confirms the model’s generalizability across different clinical settings. These findings provide a foundation for developing practical clinical decision support tools for cardiac arrest patient management.

## Supplementary Information

Below is the link to the electronic supplementary material.

Supplementary Material 1

Supplementary Material 2

## Data Availability

No datasets were generated or analysed during the current study.

## Abbreviations

MIMIC: Medical Information Mart for Intensive Care
ML: Machine learning
LR: Logistic regression
RF: Random forest
XGBoost: Extreme gradient boosting
SVM: Support vector machine
LightGBM: Light gradient boosting machine
AUC: Area under curve
ROC: The receiver operating characteristic curves
ICD-9/10: International Classification of Diseases and Ninth/Tenth Revision
PPV: Positive predictive value
NPV: Negative predictive value
BMI: Body mass index
AMI: Acute myocardial infarction
COPD: Chronic obstructive pulmonary disease
CHF: Congestive heart failure
PT: Prothrombin time
INR: International normalized ratio
BUN: Blood urea nitrogen
WBC: White blood cell count
RBC: Red blood cell count
RDW: Red cell distribution width
CKMB: Creatine kinase MB
cTnT: Cardiac troponin T
ALT: Alanine aminotransferase
SBP: Systolic blood pressure
DBP: Diastolic blood pressure
MAP: Mean arterial pressure
SpO2: Percutaneous arterial oxygen saturation
po2: Arterial blood gas oxygen partial pressure
SOFA: Sequential organ failure assessment
GCS: Glasgow coma scale
LODS: Logistic organ dysfunction system
SHAP: Shapley additive explanations
VAP: ventilator-associated pneumonia
