# Supplementary material for: Prediction of in-hospital mortality risk in cardiac arrest patients using machine learning models: a study based on the MIMIC-IV database with external validation from Yunnan University Affiliated Hospital
Source: BMC Med Inform Decis Mak. 2026 Mar 28;26:170. doi: 10.1186/s12911-026-03465-6 (PMC13185175; doi:10.1186/s12911-026-03465-6)
Supplement: Supplementary file 1 — Supplementary Material 1 [file 12911_2026_3465_MOESM1_ESM.docx]

Table S1. Baseline characteristics and survival outcomes at Yunnan University Affiliated Hospital.

| Variables | Total  (n = 52） | | Survivors  (n = 19) | | | Non-survivors  (n = 33) | | | *P-*value | | | |  |  |
| --- | --- | --- | --- | --- | --- | --- | --- | --- | --- | --- | --- | --- | --- | --- |
| age  male  BMI  **Comorbidities**  MI  hypertension  diabetes  HF  COPD  cerebral infarction  **Vital signs** | 69.2 (57.2, 80.7)  32 (61.5)  27.8 ± 4.8  21 (40.0)  27 (51.9)  20 (38.5)  16 (30.8)  1 (1.9)  11 (21.2) | | 67.4 (52.8, 81.8)  11 (57.9)  29.2 ± 5.3  7 (36.8)  13 (68.4)  8 (42.1)  7 (36.8)  0 (0)  2 (10.5) | | 69.5 (54.0, 83.2)  21 (63.6)  26.9 ± 4.4    14 (42.4)  14 (42.4)  12 (36.4)  9 (27.3)  1 (3.0)  9 (27.3) | | | | | 0.725  0.682  0.130  0.461  0.071  0.682  0.472  0.444  0.152 | | |  |  |
| temperature_mean  heart_rate_mean | 36.6 (36.2, 37.1)  82.6 (70.3, 98.1) | | 36.8 (36.5, 37.2)  79.9 (68.2, 88.1) | 36.5 (35.0, 36.9)  87.1 (73.6, 99.4) | | | | | | | 0.039  0.115 | | |  |
| sbp_mean | 117.1 (103.9, 132.1) | | 114.0 (102.3, 125.0) | 119.2 (106.3, 135.8) | | | | | | | 0.224 | | |  |
| dbp_mean | 61.4 (56.0, 68.5) | | 59.7 (55.9, 67.9) | 62.8 (56.1, 69.8) | | | | | | | 0.601 | | |  |
| mbp_mean | 81.2 (73.8, 88.1) | | 81.3 (73.3, 86.9) | 78.6 (71.2, 86.5) | | | | | | | 0.185 | | |  |
| resp_rate_mean | 20.5 (17.4, 24.0) | | 20.5 (17.4, 23.7) | 20.5 (17.4, 23.7) | | | | | | | | 0.864 | | |
| spo2_mean | 98.2 (96.9, 99.3) | | 98.5 (97.2, 99.5) | 98.2 (96.9, 99.2) | | | | | | | 0.270 | | | |
| **Laboratory results** |  | |  | | | | |  |  | | | | | |
| hematocrit_max | 34.0 (31.4, 41.4) | | 33.9 (31.1, 38.5) | 35.7 (31.7, 42.2) | | | | | | | 0.239 | | | |
| hematocrit_min | 31.4 (24.8, 37.0) | | 31.1 (23.6, 33.7) | 32.0 (26.9, 40.0) | | | | | | | 0.210 | | | |
| hemoglobin_max | 11.6 (9.8, 12.8) | | 11.5 (9.6, 12.6) | 11.7 (9.8, 13.8)) | | | | | | | 0.500 | | | |
| hemoglobin_min  wbc_max  wbc_min  platelets_max  platelets_min  glucose_max  glucose_min  bun_max  bun_min  albumin_max albumin_min  creatinine_max  creatinine_min  sodium_max  sodium_min  potassium_max  potassium_min  chloride_max  chloride_min  calcium_max  calcium_min  inr_max  inr_min  pt_max  pt_min  ph_max  ph_min  lactate_max  lactate_min  po2_max  po2_min  alt_max  alt_min  ast_max  ast_min  cTnT  ckmb  **Score system**  SOFA  GCS  LODS  Charlson | 10.0 (8.1, 11.7)  13.6 (9.8, 23.4)  10.1 (6.8, 12.6)  211.5 (157.0, 292.3)  149.5 (111.3, 214.8)  195.5 (135.8, 290.8)  129.5 (99.0, 154.0)  32.5 (17.0, 50.5)  26.0 (14.3, 43.0)  3.1 (2.6, 3.8)  3.0 (2.6, 3.7)  1.6 (0.9, 2.5)  1.3 (0.8, 2.0)  141.0(138.0, 145.0)  137.0(134.0, 140.0)  4.6 (4.2, 5.2)  3.8 (3.4, 4.3)  108.0(103.3, 112.0)  103.5 (98.3, 106.0)  8.7 (8.4, 9.1)  7.9 (7.2, 8.6)  1.5 (1.3, 2.0)  1.3 (1.1, 1.5)  16.6 (13.6, 19.5)  14.2 (12.2, 16.4)  7.4 (7.3, 7.5)  7.2 (7.1, 7.3)  3.1 (2.2, 5.9)  1.7 (1.3, 4.6)  236.5 (147.0, 285.0)  75.0 (54.0, 114.8)  76.0 (28.0, 213.0)  56.0 (25.3, 177.0)  133.5 (44.0, 272.0)  96.0 (37.3, 223.0)  0.08 (0.02, 0.27)  4.0 (2.0, 8.0)  7 (4, 11)  15 (14, 15)  7 (5, 10)  6 (3, 7) | | 9.8 (7.7, 11.2)  12.4 (10.7, 23.5)  10.6 (8.1, 11.1)  209.0 (157.0, 281.0)  148.0 (122.0, 214.0)  179.0 (133.0, 238.0)  132.0 (102.0, 143.0)  22.0 (12.0, 42.0)  19.0 (10.0, 37.0)  3.1 (2.8, 3.8)  2.8 (2.6, 3.5)  1.5 (0.8, 2.1)  1.0 (0.7, 1.4)  142.0 (138.0, 143.0)  136.0 (134.0, 140.0)  4.5 (4.2, 5.1)  3.8 (3.4, 4.0)  109.0 (105.0, 113.0)  104.0 (100.0, 107.0)  8.6 (8.3, 8.9)  7.8 (7.2, 8.3)  1.5 (1.2, 2.2)  1.2 (1.1, 1.4)  15.3 (12.4, 23.7)  13.3 (12.2, 15.5)  7.4 (7.4, 7.5)  7.3 (7.2, 7.3)  2.8 (1.7, 3.7)  1.4 (1.1, 1.7)  265.0 (149.0, 351.0)  77.0 (38.0, 110.0)  35.0 (25.0, 82.0)  31.0 (17.0, 97.0)  104.0 (50.0, 272.0)  93.0 (29.0, 134.0)  0.04 (0.02, 0.17)  3.0 (2.0, 9.0)  6 (3, 8)  15 (14, 15)  6 (4, 8)  5 (2, 6) | 10.2 (8.8, 11.9)  14.5 (9.3, 23.5)  9.3 (6.7, 14.5)  215.0 (161.5, 300.0)  151.0 (93.5, 230.5)  224.0 (137.5, 353.5)  127.0 (96.5, 193.0)  34.0 (22.0, 55.0)  27.0 (18.0, 52.0)  3.0 (2.6, 3.8)  3.0 (2.7, 3.8)  2.0 (1.1, 2.9)  1.6 (0.9, 2.1)  141.0 (138.0, 146.0)  137.0 (134.0, 139.0)  4.6 (4.3, 5.3)  3.8 (3.5, 4.6)  107.0 (103.0, 111.0)  103.0 (97.5, 106.0)  8.7 (8.4, 9.2)  8.0 (7.2, 8.7)  1.5 (1.3, 1.8)  1.3 (1.2, 1.6)  16.7 (14.5, 19.1)  14.7 (12.4, 16.6)  7.4 (7.3, 7.4)  7.2 (7.0, 7.3)  4.3 (2.8, 9.9)  3.2 (1.5, 6.2)  199.0(129.5, 282.5)  73.0 (58.0, 118.0)  90.0 (39.5, 215.0)  58.0 (25.5, 189.5)  134.0 (41.0, 318.0)  112.0 (40.5, 228.0)  0.08 (0.02, 0.45)  5.0 (2.5, 8.0)  8 (6, 13)  15 (14, 15)  8 (5, 10)  6 (4, 8) | | | | | | | 0.342  0.790  0.902  0.697  0.537  0.115  0.588  0.119  0.133  0.725  0.499  0.162  0.085  0.724  0.864  0.768  0.469  0.317  0.381  0.549  0.317  0.775  0.209  0.768  0.262  0.027  0.077  0.056  0.004  0.131  0.342  0.041  0.342  0.761  0.246  0.340  0.150  0.015  0.821  0.100  0.121 | | | |
| **Others** | |  |  | | | |  | |  | | | |  |  |
| urineoutput  ventilation  vasopressor | 1378 (626, 2122)  4.3 (1.1, 13.0)  31 (59.6) | | 1523 (875, 2165)  8.3 (2.6, 19.5)  9 (47.4) | 1130 (251, 2118)  2.1 (0.8, 9.0)  22 (66.7) | | | | | 0.183  0.019  ＜0.001 | | | |  |  |

Medical conditions were defined based on ICD-9 codes. The terms mean, minimum, or maximum refer to the average, highest, or lowest recorded level of a parameter on the first day of ICU admission. Abbreviations used are as follows: BMI, body mass index; MI, myocardial infarction; HF, heart failure; COPD, chronic obstructive pulmonary disease; WBC: white blood cell count; SpO₂, pulse oxygen saturation; po2, arterial blood gas oxygen partial pressure; INR, international normalized ratio; PT, prothrombin time; BUN, blood urea nitrogen; WBC, white blood cell count; SBP, systolic blood pressure; DBP, diastolic blood pressure; MBP, mean blood pressure; cTnT: cardiac troponin T; CKMB: creatine kinase isoenzyme MB; ALT: alanine aminotransferase; AST: aspartate aminotransferase; SOFA, Sequential Organ Failure Assessment; GCS, Glasgow Coma Scale; LODS, Logistic Organ Dysfunction System. Ventilation: Duration of ventilator use during hospitalization (measured in hours).
