# Supplementary material for: Prediction of in-hospital mortality risk in cardiac arrest patients using machine learning models: a study based on the MIMIC-IV database with external validation from Yunnan University Affiliated Hospital
Source: BMC Med Inform Decis Mak. 2026 Mar 28;26:170. doi: 10.1186/s12911-026-03465-6 (PMC13185175; doi:10.1186/s12911-026-03465-6)
Supplement: Supplementary file 2 — Supplementary Material 2 [file 12911_2026_3465_MOESM2_ESM.docx]

Table S2. Proportion of missing values for each variable in patients from the MIMIC-IV database

| Variables | % | Variables | % |
| --- | --- | --- | --- |
| age | 0 | creatinine_min | 13.7 |
| male | 0 | sodium_max | 14.5 |
| BMI | 19.7 | sodium_min | 14.5 |
| MI | 0 | potassium_max | 14.5 |
| hypertension | 0 | potassium_min | 14.5 |
| diabetes | 0 | chloride_max | 14.5 |
| HF | 0 | chloride_min | 14.5 |
| COPD | 0 | calcium_min | 17.0 |
| cerebral infarction | 0 | inr_max | 15.9 |
| temperature_mean | 5.8 | inr_min | 15.9 |
| heart_rate_mean | 5.8 | pt_max | 15.8 |
| sbp_mean | 5.8 | pt_min | 15.8 |
| dbp_mean | 5.8 | ph_max | 23.8 |
| mbp_mean | 5.8 | ph_min | 23.8 |
| resp_rate_mean | 5.8 | lactate_max | 22.9 |
| spo2_mean | 23.6 | lactate_min | 22.9 |
| hematocrit_max | 13.7 | po2_max | 23.8 |
| hematocrit_min | 13.7 | po2_min | 23.8 |
| hemoglobin_max | 13.9 | alt_max | 24.9 |
| hemoglobin_min | 13.9 | alt_min | 24.9 |
| wbc_max | 13.9 | ast_max | 24.9 |
| wbc_min | 13.9 | ast_min | 24.9 |
| platelets_max | 13.9 | cTnT | 23.4 |
| platelets_min | 13.9 | ckmb | 23.8 |
| glucose_max | 14.7 | SOFA | 12.7 |
| glucose_min | 14.7 | GCS | 11.9 |
| bun_max | 13.6 | LODS | 12.6 |
| bun_min | 13.6 | Charlson | 12.6 |
| albumin_max | 24.3 | urineoutput | 19.8 |
| albumin_min | 24.3 | ventilation | 21.1 |
| creatinine_max | 13.7 | vasopressor | 18.8 |

Table S3. Proportion of missing values for each variable in patients from the Affiliated Hospital of Yunnan University

| Variables | % | Variables | % |
| --- | --- | --- | --- |
| age | 0 | creatinine_min | 7.6 |
| male | 0 | sodium_max | 7.6 |
| BMI | 10.4 | sodium_min | 7.6 |
| MI | 0 | potassium_max | 7.6 |
| hypertension | 0 | potassium_min | 7.6 |
| diabetes | 0 | chloride_max | 7.6 |
| HF | 0 | chloride_min | 7.6 |
| COPD | 0 | calcium_min | 7.6 |
| cerebral infarction | 0 | inr_max | 13.5 |
| temperature_mean | 5.3 | inr_min | 13.5 |
| heart_rate_mean | 5.3 | pt_max | 13.5 |
| sbp_mean | 5.3 | pt_min | 13.5 |
| dbp_mean | 5.3 | ph_max | 21.2 |
| mbp_mean | 5.3 | ph_min | 21.2 |
| resp_rate_mean | 5.3 | lactate_max | 21.2 |
| spo2_mean | 5.3 | lactate_min | 21.2 |
| hematocrit_max | 7.7 | po2_max | 21.2 |
| hematocrit_min | 7.7 | po2_min | 21.2 |
| hemoglobin_max | 7.7 | alt_max | 11.8 |
| hemoglobin_min | 7.7 | alt_min | 11.8 |
| wbc_max | 7.7 | ast_max | 11.8 |
| wbc_min | 7.7 | ast_min | 11.8 |
| platelets_max | 7.7 | cTnT | 10.4 |
| platelets_min | 7.7 | ckmb | 10.4 |
| glucose_max | 7.6 | SOFA | 5.8 |
| glucose_min | 7.6 | GCS | 7.7 |
| bun_max | 7.6 | LODS | 5.8 |
| bun_min | 7.6 | Charlson | 5.8 |
| albumin_max | 11.8 | urineoutput | 7.7 |
| albumin_min | 11.8 | ventilation | 0 |
| creatinine_max | 7.6 | vasopressor | 0 |
